# Eumelanin levels in rufous feathers explain plasma testosterone levels and survival in swallows

**DOI:** 10.1002/ece3.4946

**Published:** 2019-01-30

**Authors:** Emi Arai, Masaru Hasegawa, Megumi Sato, Hidetsugu Sakai, Shosuke Ito, Kazumasa Wakamatsu

**Affiliations:** ^1^ Department of Evolutionary Studies of Biosystems Sokendai (The Graduate University for Advanced Studies) Hayama‐machi Kanagawa Japan; ^2^ Department of Biology, School of Dentistry Nihon University Tokyo Japan; ^3^ Department of Chemistry Fujita Health University School of Health Sciences Toyoake Aichi Japan

**Keywords:** pigment‐based coloration, plasma testosterone, plumage ornaments, survival, swallow, throat patch

## Abstract

Pigment‐based plumage coloration and its physiological properties have attracted many researchers to explain the evolution of such ornamental traits. These studies, however, assume the functional importance of the predominant pigment while ignoring that of other minor pigments, and few studies have focused on the composition of these pigments. Using the pheomelanin‐based plumage in two swallow species, we studied the allocation of two pigments (the predominant pigment, pheomelanin, and the minor pigment, eumelanin) in relation to physiological properties and viability in populations under a natural and sexual selection. This is indispensable for studying the evolution of pheomelanin‐based plumage coloration. Pheomelanin and eumelanin share the same pathway only during their initial stages of development, which can be a key to unravel the functional importance of pigment allocation and thus of plumage coloration. Using the barn swallow, *Hirundo rustica*, a migratory species, we found that plasma testosterone levels increased with increasing the proportion of eumelanin pigments compared with pheomelanin pigments, but not with the amount of pheomelanin pigments, during the mating period. In the Pacific swallow *Hirundo tahitica*, a nonmigratory congener, we found that, during severe winter weathers, survivors had a proportionally smaller amount of eumelanin pigments compared with pheomelanin pigments than that in nonsurvivors, but no detectable difference was found in the pheomelanin pigmentation itself. These results indicated that a minor pigment, eumelanin, matters at least in some physiological measures and viability. Because the major pigment, pheomelanin, has its own physiological properties, a combination of major and minor pigments provides multiple information to the signal receivers, potentially enhancing the signaling function of pheomelanic coloration and its diversification across habitats.

## 1 INTRODUCTION

Colorful plumage seems to have rather negative effects on survivorship (e.g., predation cost) and has thus attracted many researchers to explain its evolution and maintenance (Hill & McGraw, 2006). A possible explanation is that plumage coloration is sexually selected, if only high‐quality individuals can possess colorful plumage (Andersson, 1994; Zahavi, 1975). Melanin‐based plumage coloration is widespread in birds and is shown to be sexually selected in several species, perhaps in part because it signals the quality of birds (see McGraw, 2008; Roulin, 2016, for a recent review). Although several explanations have been proposed for the mechanisms enforcing the honest association (e.g., resource trade‐off, pleiotropy), the relative importance of each explanation is still debated (Ducrest, Keller, & Roulin, 2008; Roulin, 2016).

Melanin‐based coloration can be subdivided into yellow–reddish pheomelanic and gray–blackish eumelanic coloration (Ito & Wakamatsu, 2003), based on the relative abundance of two pigments, pheomelanin and eumelanin (Ito et al., 2011). Both melanin pigments are derived from the common precursor dopaquinone, which is produced from tyrosine by the enzyme tyrosinase (Hearing, 2011; Ito & Wakamatsu, 2008). While the melanocyte‐stimulating hormone (MSH) leads the production of eumelanin pigments, the cysteine availability determines pheomelanin levels produced in melanosomes (Ito & Wakamatsu, 2011). MSH exerts pleiotropic effects on many physiological processes by binding to five melanocortin receptors, MC 1–5R, and this biding is hypothesized to be the cause of the association between melanin‐based coloration and physiological and behavioral traits (Ducrest et al., 2008), together with the epistatic mechanism (i.e., melanin‐based coloration itself influences body condition; Roulin, 2016; Safran, Adelman, McGraw, & Hau, 2008)*.*

Pheomelanic coloration has recently attracted particular attention, because pheomelanin pigments are phototoxic, and thus, their evolution is sometimes considered as “an accident of nature” (Galván, Ghanem, & Møller, 2012; reviewed in Napolitano, Panzella, Monfrecola, & d'Ischia, 2014; but see Galván & Wakamatsu, 2016, for pigments in the feathers). Several studies have focused on pheomelanic coloration and found its relationship with important physiological properties (Gasparini et al., 2009; Grunst, Salgado‐Ortiz, Rotenberry, & Grunst, 2014; Piault, Gasparini, Bize, Jenni‐Eiermann, & Roulin, 2009; Roulin, Almasi, Meichtry‐Stier, & Jenni, 2011; Saino, Canova et al., 2013), viability (Galván & Møller, 2013; Hasegawa, Arai, Watanabe, & Nakamura, 2014a; Saino, Romano, Rubolini, Ambrosini et al., 2013), and life history strategies (Emaresi et al., 2013), but only a few studies have directly tested the importance of pheomelanin pigmentation (e.g., the relationship between pheomelanin levels of males and breeding onset: Arai, Hasegawa, Nakamura, & Wakamatsu, 2015, the relationship between pheomelanin levels of nestlings and oxidative status: Arai et al., 2017). This is unfortunate, because pheomelanic coloration is in fact affected by pheomelanin and eumelanin pigments (i.e., colorful feathers include both pheomelanin and eumelanin pigments; McGraw, Safran, & Wakamatsu, 2005; Saino, Romano, Rubolini, Teplitsky et al., 2013) as well as several post‐molting processes (e.g., wearing, stains, and so on; Hasegawa, Arai, Watanabe, & Nakamura, 2008; Safran, McGraw, Wilkins, Hubbard, & Marling, 2010; reviewed in Montgomerie, 2006). Therefore, it remains unclear whether the observed relationship between pheomelanic plumage coloration and physiological properties is caused by the major pigment, pheomelanin, or is affected by additional constituents of feathers, which is indispensable when uncovering how and why pheomelanic plumage coloration evolved, is maintained, and has diversified across habitats.

Plasma testosterone level is thought to be linked to male melanin‐based plumage ornamentation, reproductive parameters, and survivorship (reviewed in Wingfield, Lynn, & Soma, 2001). Using the North American barn swallow, *Hirundo rustica erythrogaster*, Safran et al. (2008) formally showed the causal link between male pheomelanic plumage coloration (i.e., pheomelanin‐rich coloration) and plasma testosterone levels, because enhanced male plumage coloration (i.e., males with darkened plumage) increased plasma testosterone levels, perhaps mediated by social interaction (also see Levin et al., 2016; Rubenstein & Hauber, 2008). Eikenaar, Whitham, Komdeur, Velde, and Moore (2011) also found a positive correlation between male pheomelanic plumage coloration and plasma testosterone levels in the North American subspecies (i.e., darker males showed higher testosterone levels). In addition, as predicted by the positive relationship between plasma testosterone levels and aggressiveness, colorful (or darker) male swallows hold superior territories in the North American (Wilkins, Shizuka, Joseph, Hubbard, & Safran, 2015) and Asian barn swallows *Hirundo rustica gutturalis* (Hasegawa, Arai, Watanabe, & Nakamura, 2014b). Hasegawa et al. (2014b) also found a negative correlation between male pheomelanic coloration and paternal care, which was also predicted by a negative link between plasma testosterone level and paternal care. However, all these studies focused on plumage coloration rather than pigmentation, and thus, actual importance of pheomelanin, eumelanin, and other components (e.g., post‐molting process such as wear; e.g., Arai et al., 2015) was unclear. Finally, although plasma testosterone level would deteriorate the survival of well‐ornamented individuals during the nonbreeding, wintering period in which available resources for somatic maintenance are limited (Koren et al., 2012), a direct comparison of melanin composition between survivors and nonsurvivors is lacking.

Wintering periods are energetically demanding (Koren et al., 2012). In 2016, Amami Oshima Island, Japan, experienced a rare severe winter. It was snowfall for the first time in 115 years in this subtropical region with the lowest air temperature ever reported and 188% higher precipitation than the average in this region (Japan Meteorological Agency, 2016). The Pacific swallow, *Hirundo tahitica*, is a nonmigratory resident bird and can forage only when weather conditions allow flying insects to be active. After the severe weather, it was found that many Pacific swallows died (Hasegawa & Arai, 2017). Because the Pacific swallow would not be capable of avoiding inclement weather by long‐distance dispersal (Turner & Rose, 1994, p 173), this meteorological event provided a rare opportunity to study the relationship between melanin pigmentation and survival without confounded by differential dispersal (i.e., surviving and nonsurviving individuals can be directly compared). This contrasts with migratory birds in which dispersal and nonsurvival are hardly distinguished (e.g., see Arai, Hasegawa, & Nakamura, 2009 for the barn swallow).

**Figure 1:**
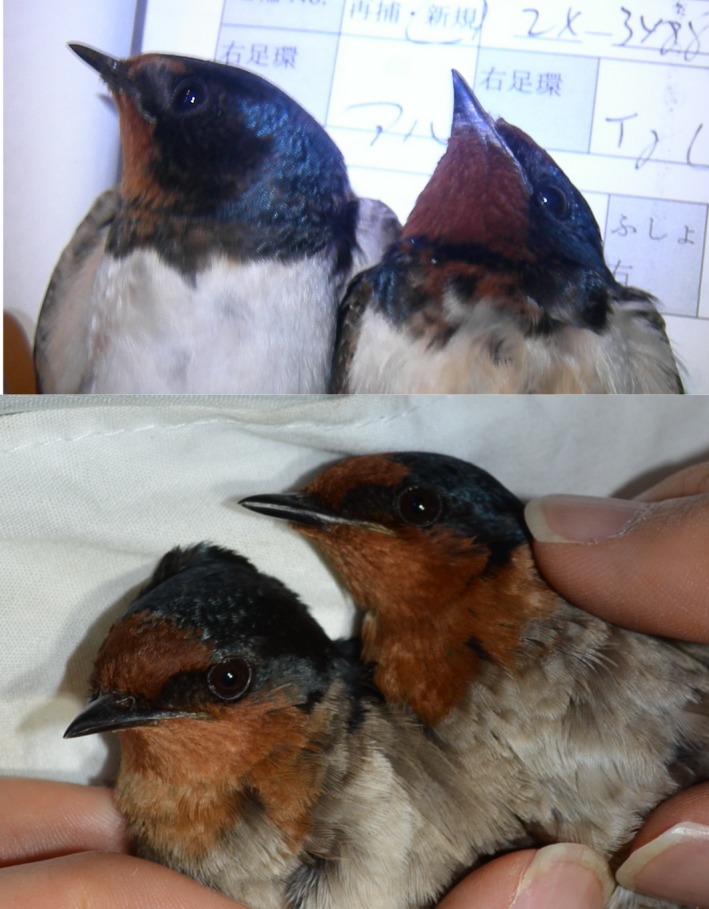
Throat patches of the barn swallow* Hirundo rustica* (upper panel: right, male; left, female) and the Pacific swallow *Hirundo tahitica *(lower panel: right, male; left, female)

Here, we studied whether melanin constituents (i.e., pheomelanin and eumelanin) can be linked to physiology and survival using two swallow species, the barn swallow, *H. rustica *(subspecies *H. r. gutturalis*), and the Pacific swallow, *H. tahitica*, both of which have a pheomelanic throat patch (Figure 1). Because the barn swallow is a migratory bird species, its mating period is often confined shortly after spring migration (e.g., Arai et al., 2009), making the barn swallow well suited to study behaviors and physiological properties during the mating period (in contrast to nonmigratory species, which tend to maintain their partnerships continuously more often than migratory species: reviewed in Ens, Choudhury, & Black, 1996). In fact, this is a model species of sexual selection (Møller, 1994; Turner, 2006), and the pheomelanic coloration has been shown to be condition‐dependent and sexually selected in some populations including Japanese barn swallows (e.g., Arai et al., 2015; Hasegawa, Arai, Watanabe, & Nakamura, 2010; reviewed in Romano, Constanzo, Rubolini, Saino, & Møller, 2017). Then, we predicted that pheomelanin pigmentation, which is the main pigment of the throat patch (Arai et al., 2015), is linked to the level of plasma testosterone during mating periods based on previous studies that showed a positive link between pheomelanic coloration and plasma testosterone levels (see above). If pheomelanic coloration is associated with plasma testosterone levels due to the physiological or mechanistic properties of pheomelanin pigmentation together with social reinforcement, pheomelanin pigmentation, rather than other constituents of pheomelanic coloration (e.g., eumelanin), would be positively associated with plasma testosterone levels. In the Pacific swallow, we predicted that pheomelanin pigmentation would be linked to survival if this trait is related to testosterone level (e.g., see Koren et al., 2012, for the negative link between testosterone level and survival in the nonbreeding period in the house sparrow). If high pheomelanin pigmentation is costly due to its possible link with testosterone levels, birds with less pheomelanin pigmentations should survive better than others during severe weathers. We found that these predictions were not supported and that eumelanin pigmentation, rather than pheomelanin, explained plasma testosterone levels during the mating period in barn swallows and survival of wintering Pacific swallows. We discussed the ecological and evolutionary implications of the observed pattern.

## 2 MATERIALS AND METHODS

### 2.1 Field study of barn swallows

Field surveys were conducted in a residential area of Hayama‐machi, Kanagawa Prefecture, Japan (35°160′N, 139°350′E, alt 43 m), from March to May 2015 and 2016. In this area, barn swallows nest under the eaves of houses along streets (Hasegawa, Arai, Ito, & Wakamatsu, 2016). After male arrival, territory ownership (and its turnover) was observed using binoculars. Sweep nets were used to capture adult males during the mating period while they were roosting at night. The sex of each individual was determined based on the lengths (and shape) of the outermost tail feathers and of the occurrence of cloacal protuberances (Turner, 2006). At capture, we marked each bird with a unique combination of two or three colored rings (Arai et al., 2009) and collected blood samples from the brachial vein using heparinized capillary tubes within five minutes of capture. Blood samples were centrifuged immediately to separate plasma from red blood cells and other sediments. The samples were then stored in an ice box until returning to the laboratory (within 2 hr), where they were stored at −80°C until analysis. After collecting blood samples, we measured body mass (to the nearest 0.1 g), wing length, keel length, tail length, size of the white tail spots (the sum of the lengths of the white spots of the two outermost right tail feathers; all measurements to 0.01 mm precision), and throat patch area (see below). Body condition was then estimated as a residual of body mass on keel length (linear model: coefficient ± *SE* = 0.73 ± 0.33, *F*_1,23_ = 4.81, *p* = 0.039). We also collected approximately 10–20 throat feathers to quantify the pheomelanin and eumelanin contents. Detailed information on the measurements can be found in previous publications (Hasegawa & Arai, 2013; Hasegawa et al., 2010).

The rufous throat patch size was defined as the size of the area covered by rufous throat feathers (Hasegawa et al., 2010). This was measured by placing a transparent plastic sheet over the throat region, ensuring that the feathers were lying flat in their natural position, and tracing the edges of the patch onto the sheet using a marker pen (Lendvai, Kis, Szekely, & Cuthill, 2004). We then scanned the sheet and measured the area of the patch (mm^2^) using the Scion Image software (Scion Corporation, Frederick, MD, USA). The throat patch of each bird was traced twice, and the mean of the two measurements was used for further analyses (repeatability = 0.87 and 0.86 for 2015 and 2016, respectively; Lessells & Boag, 1987).

### 2.2 Field study of pacific swallows

Field surveys were conducted on Amami Oshima Island, Kagoshima Prefecture, Japan (28°22′N, 129°29′E; total area 712.35 km^2^), from 28 January to 6 February 2016. Pacific swallows inhabit this island throughout the year, and no barn swallows are wintering or breeding there. As in barn swallows, Pacific swallows (Figure 1) were captured in sweep nets while roosting at night. In these areas, swallows nest and roost inside buildings, under bridges, or under the eaves of houses (see fig. 2 in Mikuriya, 1968). At capture, we marked each bird with metal rings with unique numbers (AC Hughes Ltd., Middlesex, UK), and collected blood samples from their brachial veins using heparinized capillary tubes (for sex identification; see Hasegawa & Arai, 2017, for detailed information). MH measured wing length, keel length, and outermost tail length (nearest 0.01 mm), as measured in the barn swallow (e.g., Hasegawa et al., 2010; also see above). Throat patch size was not used in the current study, because this trait was difficult to measure on dead birds and a direct comparison with live birds was deemed impractical (Hasegawa & Arai, 2017). However, all Pacific swallows had fully developed throat patches with no black breast band (Turner & Rose, 1994; also see Figure 1), and thus, their variation would be small compared to those of barn swallows. Ten feathers were plucked from the throat feathers to determine the pigmentation afterword. We confirmed that all these birds had completed the molt at capture.

During the study period, we also searched for dead birds, which were found under or inside nests. As was done for live birds, MH measured wing length, keel length, tail length of the dead birds. Although the measurements of dead birds can sometimes introduce a measurement bias due to skin shrinkage or other artifacts, such a bias should be negligible in swallows (Brown & Brown, 1998, 1999). Ten feathers were plucked from the throat feathers to determine the pigmentation afterword (see above). All dead birds were placed in plastic bags and stored at −20°C until being sent to the laboratory, where they were stored at −80°C. In the laboratory, we took some feathers or tissues from these birds for sex identification (see Hasegawa & Arai, 2017 for detailed methods). As for live birds, all dead birds had completed the molt.

### 2.3 Testosterone ELISA

We separately analyzed samples collected in 2015 and 2016. Blood plasma samples in 2015 were analyzed for testosterone in duplicate by ELISA (Cayman #582701). For samples of very small volume per tube (<3 µl, three samples), we could not adequately separate sediments from the plasma sample, and thus, we discarded these samples (Hasegawa, Arai, Sato, & Sakai, 2017). Mean blood plasma volume per tube of the remaining samples was 11 µl (range: 5–27.5 µl) in 2015. We calculated plasma testosterone concentration using the standard curve, which was well fitted to the data (*r* = 0.99). Sensitivity (80% maximum binding) and midpoint (50%) were 14.01 and 67.93 pg/ml, respectively. As recommended by the manufacturer's protocol, any sample outside the assay range, 3.9–500 pg/ml, was discarded (six such samples, all below 3.9 pg/ml). The remaining samples were run in a single assay (using split with two mounts), and intra‐assay variation, calculated as the mean coefficient of variance of the duplicates, was 13% in the data set of 2015 (with repeatability = 0.99, *n* = 33, *F* = 477.23, *p < *0.0001). Because duplicates showed highly repeatable values, and because we lost some samples due to small volume per tube, we did a single analysis (i.e., did not duplicate) for the samples collected in 2016 to prevent further sample loss. In 2016, mean blood plasma volume per tube was 20 µl (range: 7–33 µl) and standard curve was well fitted to the data (*r* = 0.99). Sensitivity (80% maximum binding) and midpoint (50%) were 7.97 and 56.22 pg/ml, respectively, in 2016. No samples fell outside the assay range of 3.9–500 pg/ml in 2016. Cross‐reactivity of the assay is generally low, except for 5a‐dihydrotestosterone (5a‐DHT: 27.4%) as shown in the manufacturer's protocol, although the 5a‐dihydrotestosterone concentration is very low in barn swallows (e.g., undetectable in 44% of breeding males; Saino & Møller, 1994). Because previous analysis indicated that testosterone levels drop after the mating period (Hasegawa et al., 2017), we used solely data obtained during mating period.

### 2.4 Pigment analysis

The concentrations of melanin pigments were determined by high‐performance liquid chromatography (HPLC) according to the method described previously (Ito et al., 2011). The HPLC system consisted of a JASCO 880‐PU pump (JASCO Co., Tokyo, Japan), a Shiseido C18 column (Capcell Pak MG; 4.6 × 250 mm; 5 μm particle size; Shiseido, Tokyo, Japan), and a JASCO UV‐970 UV/VIS detector at 269 nm. The mobile phase was 0.1 M potassium phosphate buffer (pH 2.1):methanol = 99:1 (v/v). Analyses were performed at a flow rate of 0.7 ml/min at 45°C. After removing the eumelanic proximate part from each feather sample, the remaining pheomelanic parts were put into a 10‐ml screw (Te)‐capped conical glass test tube, to which 40 μl water, 150 μl 1 M K_2_CO_3_, and 10 μl 30% H_2_O_2_ (final concentration: 1.5%) were added. The mixture was mixed vigorously at 25°C (range, 24–26) for 20 hr. The residual H_2_O_2_ was decomposed by adding 20 μl 10% Na_2_SO_3_, and the mixture was then acidified with 56 μl 6 M HCl. After vortexing, the reaction mixture was transferred to a 1.5‐ml Eppendorf tube and centrifuged at 10,000 *g* for 1 min, and an aliquot (80 μl) of the supernatant was directly injected into the HPLC system. Given the amount of dilution used for the current study, amounts of degradation product from pheomelanin, thiazole‐2,4,5‐tricarboxylic acid (TTCA), and that of eumelanin, pyrrole‐2,3,5‐tricarboxylic acid (PTCA), were measured (Ito et al., 2011), which were divided by the number of feathers to calculate each product per feather. We used TTCA as a measure of pheomelanin and PTCA/TTCA as a measure of the eumelanin (more precisely, eumelanin pigmentation in relation to pheomelanin pigmentation; Ito et al., 2011, Wakamatsu, Nakanishi, Miyazaki, Kolbe, & Ito, 2012). We used the latter measure instead of PTCA itself because a small amount of PTCA can be produced from the degradation of pheomelanin pigment (d'Ischia et al., 2013). In other words, PTCA/TTCA can be a better measure of eumelanin pigmentation than PTCA because melanins from red throat feathers could be copolymers of both melanins [Ito & Wakamatsu, 2003; Ito et al., 2013; Wakamatsu et al., 2012; although we could estimate the coefficient of PTCA after log transformation; i.e., log(PTCA/TTCA) equals log(PTCA) − log(TTCA), and thus, this is not statistically problematic; see Supporting Information Appendices S1 and S2 for the alternative analysis using the residuals of a regression of the log(PTCA) on log(TTCA) instead of log(PTCA/TTCA)].

### 2.5 Statistics

Plasma testosterone level, TTCA, and PTCA/TTCA were log‐transformed before analysis, as in the previous studies (e.g., Hasegawa et al., 2016, 2017). To study the relationship between pigmentation and testosterone level, we used a linear model (LM) with plasma testosterone level as a response variable. To account for any potential confounding effects of study year and other male ornaments (i.e., tail length, the size of white tail spots, and throat patch size; Hasegawa et al., 2010), these were used as covariates. We standardized each variable to a mean of zero and unit variance before analysis.

To study selection on wintering Pacific swallows, we used multivariable logistic regression as in preceding studies (e.g., Grant & Grant, 2013; Hasegawa & Arai, 2017). From this analysis, we studied the linear selection gradient (*β*), which is direct selection on each trait (Janzen & Stern, 1998; Lande & Arnold, 1983), based on the slope coefficient, the proportion of live birds in all samples, and the predicted survival of each individual (see Janzen & Stern, 1998 for detailed calculations). For this purpose, we standardized each log‐transformed value to a mean of zero and unit variance (Hasegawa & Arai, 2017). Because flight apparatuses (i.e., wing length and tail length) were shown to affect survival of wintering Pacific swallows (Hasegawa & Arai, 2017), we included these variables as covariates.

Lastly, we studied sex‐ and species‐specific expression of pigmentation. For this purpose, we analyzed PTCA in relation to TTCA, sex, and species using a linear model. By including TTCA as a covariate, we could control the positive association between PTCA and TTCA (and then pheomelanin‐derived small amount of PTCA, which should linearly increase with TTCA; d'Ischia et al., 2013). By this approach, we can study how PTCA changes with TTCA in relation to sex and species (and this represents a better approach than using PTCA/TTCA against TTCA here).

When we tested for multicollinearity among variables using the variance inflation factor (VIF; a VIF of >2.5 would be problematic; Allison, 1999; Graham, 2003; see also Allison, 2012), we mostly found very low VIF values (see tables for each statistics), indicating that multicollinearity might have few effects on the estimates. When max VIF exceeds 2.5, which can affect the conclusion in some cases, and thus, we tested and confirmed that an additional model excluding predictors with a large VIF value yielded qualitatively similar results. All statistical analyses were conducted with R 3.3.0 (R Core Team, 2016).

## 3 RESULTS

### 3.1 Testosterone and pigmentation levels in the barn swallow

**Figure 2:**
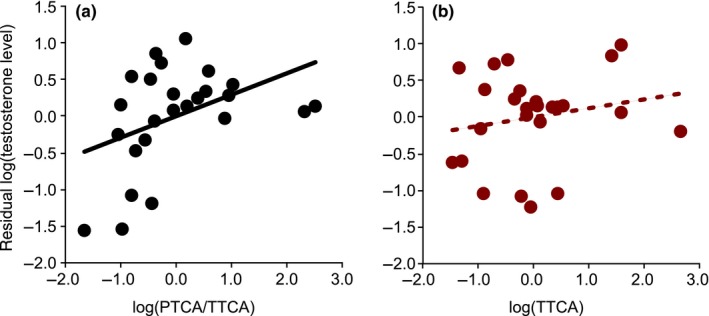
Plasma testosterone level increased with (a) eumelanin:pheomelanin ratio measured as log(PTCA/TTCA), but not with (b) pheomelanin pigmentation, measured as log(TTCA), in the mating period of *Hirundo rustica *after controlling for covariates*. *Simple regression lines are shown (see Table 1 for formal statistics). log(PTCA/TTCA) and log(TTCA) were standardized with zero mean and unit variance (the former: mean ± *SD* = −2.39 ± 0.22: the latter: 4.09 ± 0.19). PTCA: pyrrole‐2,3,5‐tricarboxylic acid; TTCA: thiazole‐2,4,5‐tricarboxylic acid

The measure of eumelanin pigmentation, log(PTCA/TTCA), was significantly positively related to plasma testosterone levels in male barn swallows during the mating period (Table 1; Figure 2a; also see Appendix S1). Pheomelanin pigmentation, measured as log(TTCA), and other measures of male ornaments were not significantly related to plasma testosterone levels (Table 1; Figure 2b). When excluding two outliers (i.e., >2SD: Figure 2a), log(PTCA/TTCA) remained significant (*F* = 8.67, *p* < 0.01), while other variables remained nonsignificant (*F* < 1.82, *p* > 0.19). When we added body condition as another explanatory variable, plasma testosterone level marginally increased with decreasing body condition (coefficient ± SE = −0.32 ± 0.15, *F = *4.26, *p* = 0.055; note that similar relationships were found for other variables, that is, significant log(PTCA/TTCA), *F* = 7.88, *p* = 0.01, and nonsignificant remaining variables; *F* < 2.83, *p* > 0.11). Body condition was not significantly related to study year, pigmentation, and other male ornaments in the current data set, both in univariable and in multivariable linear models (*p* > 0.10; data not shown).

**Table 1:** Linear model explaining log(plasma testosterone level) in relation to eumelanin:pheomelanin ratio, measured as log(PTCA/TTCA), and pheomelanin pigmentation, measured as log(TTCA), in male barn swallows (*n* = 25)

	Coefficient ± *SE*	*F*	*p*
log(PTCA/TTCA)	**0.39 ± 0.17**	**5.44**	**0.032**
log(TTCA)	0.16 ± 0.17	0.96	0.34
Tail length	0.06 ± 0.17	0.13	0.72
Size of white tail spots	0.23 ± 0.19	1.44	0.25
Throat patch area	0.18 ± 0.15	1.40	0.25
Study year (2016–2015)	0.08 ± 0.32	0.06	0.82

Notes

Each variable was standardized to mean zero and unit variance after log transformation. Significant test result (*p* < 0.05) is indicated in bold.

Max VIF = 1.70.

Note that log(PTCA/TTCA) equals log(PTCA) − log(TTCA).

PTCA: pyrrole‐2,3,5‐tricarboxylic acid; TTCA: thiazole‐2,4,5‐tricarboxylic acid.

### 3.2 Survival and pigmentation levels in the pacific swallow

**Figure 3:**
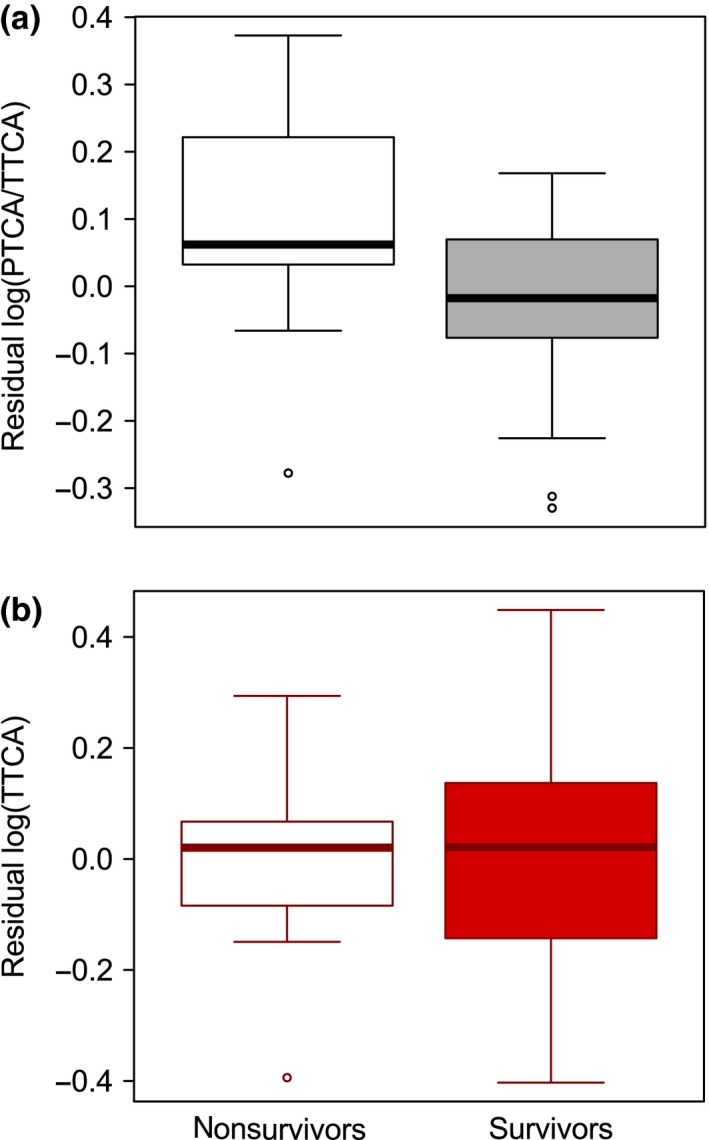
Survivors had (a) low eumelanin:pheomelanin ratio measured as log(PTCA/TTCA) but had (b) similar pheomelanin pigmentation measured as log(TTCA) than that of nonsurvivors in the nonbreeding period of *Hirundo tahitica* after controlling for covariates listed in Table 2. The horizontal bar in each boxplot indicates the median, and the box shows the first and third quartiles of data. The whiskers range from the lowest to the highest data points within 1.5× interquartile range of the lower and upper quartiles, respectively. The data point beyond the range of the whiskers represents an outlier. PTCA: pyrrole‐2,3,5‐tricarboxylic acid; TTCA: thiazole‐2,4,5‐tricarboxylic acid

After statistically controlling for flight apparatus (wing and tail length), which were the main predictors of survival (Hasegawa & Arai, 2017), eumelanin but not pheomelanin pigmentation explained the survival of Pacific swallows (Table 2; also see Appendix S2), with less eumelanic swallows surviving better than others (Figure 3).

**Table 2:** General linear model with a binomial distribution explaining survival in relation to wing length, tail length, and pigmentation (i.e., pheomelanin pigmentation, measured as log(TTCA) and eumelanin:pheomelanin ratio, measured as log(PTCA/TTCA) in nonbreeding Pacific swallows (sample sizes: males, *n*_survivors_ = 16, *n*_nonsurvivors_ = 5; females, *n*_survivors_ = 19, *n*_nonsurvivors_ = 4; *n*_total_ = 44)

	Coefficient ± *SE*	*χ* ^2^	*p*	*β*
log(wing length)	**2.69 ± 0.99**	**17.78**	**<0.0001**	**0.24**
log(tail length)	**−1.71 ± 0.89**	**5.40**	**0.02**	**−0.15**
log(PTCA/TTCA)	**−1.52 ± 0.79**	**6.52**	**0.01**	**−0.13**
log(TTCA)	−0.03 ± 0.72	0.00	0.97	0.00

Notes

Each variable was standardized to mean zero and unit variance after log transformation.

Selection gradients (*β*) were measured as the averaged gradient vector (Janzen & Stern, 1998).

Including sex and its interaction with main terms did not change the results qualitatively (i.e., significant and nonsignificant terms remain unchanged). Significant test result (*p* < 0.05) is indicated in bold.

Max VIF = 2.94 (though the variable with highest VIF, tail length, did not change the result qualitatively, in which Max VIF reduced to 1.77).

Note that log(PTCA/TTCA) equals log(PTCA) − log(TTCA).

PTCA: pyrrole‐2,3,5‐tricarboxylic acid; TTCA: thiazole‐2,4,5‐tricarboxylic acid.

### 3.3 Sex, species, and pigments

**Figure 4:**
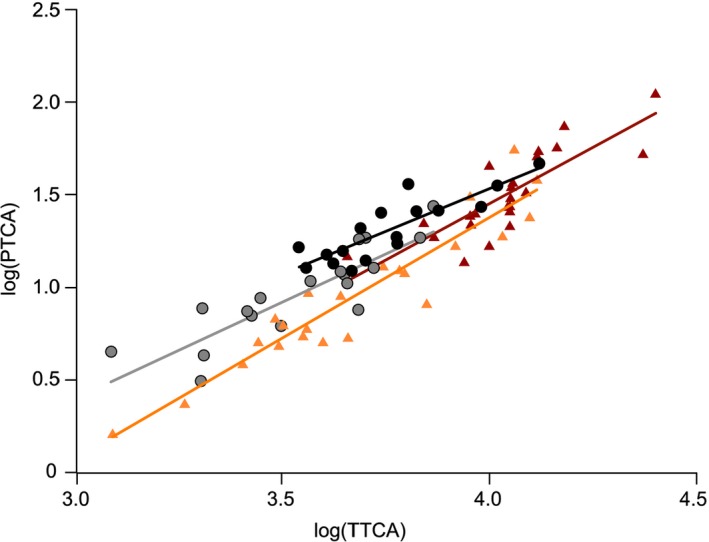
Sex and species dependency of log(PTCA) in relation to log(TTCA). Red and orange triangles indicate male and female *Hirundo rustica*, respectively. Black and gray circles indicate male and female *Hirundo tahitica*, respectively. Simple regression lines for each category are shown (see Table 3 for formal statistics). PTCA: pyrrole‐2,3,5‐tricarboxylic acid; TTCA: thiazole‐2,4,5‐tricarboxylic acid

In 2016, we captured both *H. rustica* and *H. tahitica*. After statistically controlling for measure of pheomelanin pigmentation levels (i.e., log(TTCA)), measure of eumelanin pigmentation levels (log(PTCA)) was significantly explained by sex and species (Table 3): After controlling for positive relationship with log(TTCA), males and *H. tahitica* had higher eumelanin levels than females and *H. rustica*, respectively (Figure 4).

**Table 3:** Linear model explaining log(PTCA) in relation to log(TTCA), species, and sex in genus *Hirundo *(*n*_total_ = 82; males: *n*_tahitica_ = 17, *n*_rustica_ = 23; females: *n*_tahitica_ = 19, *n*_rustica_ = 23)

	Coefficient ± SE	*F*	*p*
log(TTCA)	**0.91 ± 0.05**	**314.76**	**<0.0001**
Species (*H. tahitica* = 1)	**0.43 ± 0.09**	**25.35**	**<0.0001**
Sex (male = 1)	**0.28 ± 0.09**	**9.07**	**<0.01**

Notes

Each continuous variable was standardized to mean zero and unit variance after log transformation.

Significant test results (*p* < 0.05) are indicated in bold.

Max VIF = 1.80.

PTCA: pyrrole‐2,3,5‐tricarboxylic acid; TTCA: thiazole‐2,4,5‐tricarboxylic acid.

## 4 DISCUSSION

The main finding of the current study is that the eumelanin, not pheomelanin pigmentation levels, consistently explained a physiological property, plasma testosterone level, during the mating period in barn swallows and survival of wintering Pacific swallows. Thus, some properties of pheomelanic plumage coloration, that is, its link with testosterone and survivorship, are at least in part due to higher amount of eumelanin in relation to pheomelanin pigmentation. Because some previous studies have demonstrated that pheomelanin pigmentation is linked to other physiological properties (e.g., resistance to oxidative stress: Roulin et al., 2011; antioxidant level: Arai et al., 2017), both constituents, pheomelanin and eumelanin, jointly provide multiple information of individual quality.

Theoretically, the positive link between eumelanin deposition and plasma testosterone level is predictable, because the eumelanin production pathway is linked to testosterones via melanocortin systems (Ducrest et al., 2008). For example, melanocortin (i.e., melanin‐stimulating hormones α‐, β‐, and γ‐MSHs and adrenocorticotropin hormone, ACTH) enhances the production of sexual hormones, male sexual motivation, and performance as well as eumelanin pigment production (Ducrest et al., 2008). For this reason, even in pheomelanin‐predominated feathers, eumelanin levels (and thus its relative investment with pheomelanin pigments) may reflect individual physiological aspect, and thus, males exhibit more eumelanic feathers only when they can endure high testosterone levels and the associated costs (e.g., low survival: Koren et al., 2012; immunosuppressive effect: Saino, Møller, & Bolzerna, 1995, but see Owen, Nelson, & Clayton, 2010; reviewed in Wingfield et al., 2001). It is at first surprising to see that a small portion of eumelanin pigmentation in pheomelanic feathers honestly indicates individual physiological properties, because all the dorsal feathers are uniformly eumelanic in the focal species. A possible explanation is that eumelanin pigments accompanied by pheomelanin pigments augment the signal honesty. Eumelanogenesis and pheomelanogenesis share the early stage of their production pathway; both are derived from a common precursor, dopaquinone, which is produced from L‐tyrosine by tyrosinase and controlled antagonistically thereafter (Ducrest et al., 2008; Ito & Wakamatsu, 2008; Wakamatsu & Ito, 2002). Dopaquinone is a highly reactive intermediate, and it undergoes eumelanogenesis in the absence of sulfhydryl compounds (Ito & Wakamatsu, 2008). Therefore, birds with eumelanin‐rich pheomelanic feathers might have more l‐tyrosine or a higher level of tyrosinase activity than those of birds with less eumelanin. Because testosterone influences tyrosinase stimulation (e.g., male willow ptarmigan *Lagopus lagopus lagopus*; Stokkan, 1979), the difference in tyrosinase activity via testosterone might also link between eumelanin levels in pheomelanic plumage and physiological properties, which are not so related to pheomelanin pigment. The reverse pattern was found in Galván, Wakamatsu, Camarero, Mateo, and Alonso‐Alvarez (2015) in which pheomelanin pigmentation in eumelanin‐predominated feathers (black breast stripe in the great tit) signal individual quality via a trade‐off of antioxidants (i.e., sulfhydryl compounds) between pheomelanin production and somatic maintenance. Even such a small component of feathers may be the target of selection particularly when it can be a composite trait with other indicator traits (e.g., see Arai et al., 2017 for patch size and coloration). Social interaction further augments the relationship between the target of selection and testosterone level (Safran et al., 2008), though relative importance of eumelanin and pheomelanin pigmentation is unclear. As predicted by the pheomelanic coloration–testosterone link, the positive pheomelanic coloration–territory link was sometimes demonstrated (e.g., Hasegawa et al., 2014b; Wilkins et al., 2015). At the first glance, the association appears to be driven by pheomelanin pigmentation but might be explained by eumelanin pigmentation. This is consistent with previous studies showing the function of melanocortin to eumelanin pigmentation and testosterone (Ducrest et al., 2008) or social reinforcement on hormone and pigmentation (e.g., McGraw, Dale, & Mackillop, 2003, reviewed in Kimball, 2006; see also above). Rather than assuming that the predominant pigmentation plays a role for the link between plumage coloration and physiological traits, we should carefully argue the role of each pigment component (and their coexistence) while the theoretical background is taken into account.

The winter survival in relation to eumelanin pigments in the Pacific swallow is also consistent with the theoretical expectation of the effects of testosterone levels on survival (Ketterson & Nolan, 1992; Koren et al., 2012; Wingfield et al., 2001). Because of their close phylogenetic relationship (e.g., Sheldon, Whittingham, Moyle, Slikas, & Winkler, 2005), Pacific swallows might share a similar physiological link between eumelanin/pheomelanin pigmentation and testosterone levels with barn swallows. However, the testosterone‐mediated viability cost is not the sole explanation for the observed pattern. For example, eumelanin and pheomelanin have their own physiological costs, because several other physiological (and behavioral) properties are linked to these pigments (d'Ischia et al., 2013; Ducrest et al., 2008; Roulin et al., 2011). Future studies should disentangle these alternative but not mutually exclusive explanations. In any cases, the current study showed that proportionally more eumelanic birds had survival costs during severe winter period, which should not be ignored when studying the evolution of pheomelanic coloration.

In conclusion, we found that a minor pigment, that is, eumelanin pigment level in pheomelanic plumage, could indicate some physiological properties together with the major pigment, pheomelanin, rather than mere by‐product of major pigments. Because pheomelanin pigmentation has its own properties (e.g., glutathione‐mediated antioxidant capacity: Arai et al., 2017), different pigment components and associated physiological properties might matter in different context of social interactions (e.g., highly aggressive, testosterone‐rich males might be favored in a male‐male contest but be disfavored by potential social mates because they exhibit reduced paternal care; Wingfield, Hegner, Dufty, & Ball, 1990). Such differential social interactions and the resulting selection, if any, can explain why Pacific swallows, a nonmigratory species relying more on their territory, have a proportionally higher amount of eumelanin and thus more blackish coloration than their migratory congeners, barn swallows. This would also explain why males who defend a territory have proportionally higher amounts of eumelanin than females. Although previous correlational and experimental studies regard color as a continuum of colorful to drab (or dark to light in the barn swallow; e.g., Eikenaar et al., 2011; Hasegawa & Arai, 2016; Jenkins, Vitousek, & Safran, 2013; Saino, Romano, Rubolini, Teplitsky et al., 2013), future studies should carefully argue the multidirectional properties of plumage coloration, its pigment composition, and their perception by the focal species (see McGraw et al., 2005 for the effect of eumelanin on plumage coloration), to explain the evolution of plumage coloration and its divergence. Experimental validation of eumelanin‐ and pheomelanin‐dependent hormonal expression should also be shown. Without considering how feather color is composed, plumage color studies might fail or misunderstand the function, evolution, and diversification of plumage coloration.

## CONFLICT OF INTEREST

None declared.

## AUTHOR CONTRIBUTIONS

MA and EA conceived the ideas, designed methodology, collected field data, and wrote the drafts. SI and KW helped EA to do pigment analysis. MS and HS helped MA and EA to do ELISA. All authors contributed to the drafts and gave final approval for publication.

## Data Availability

Data are available from Dryad Digital Repository, https://doi.org/10.5061/dryad.b433hv8.
