# Supplementary material for: Eumelanin levels in rufous feathers explain plasma testosterone levels and survival in swallows
Source: Ecol Evol. 2019 Jan 30;9(5):2755–64. doi: 10.1002/ece3.4946 (PMC6405925; doi:10.1002/ece3.4946)
Supplement: Supplementary file 1 [file ECE3-9-2755-s001.docx]

**Appendix 1.** Linear model explaining log(plasma testosterone level) in relation to eumelanin pigmentation, measured as residual log(PTCA), and pheomelanin pigmentation, measured as log(TTCA) in male barn swallows (*n* = 25).

| Coefficient ± SE *F* *P* |
| --- |
| residual log(PTCA)† **0.35 ± 0.15 5.44 0.032**  log(TTCA) 0.01 ± 0.16 0.01 0.93  Tail length 0.06 ± 0.17 0.06 0.81  Size of white tail spots 0.23 ± 0.19 1.60 0.22  Throat patch area 0.18 ± 0.15 1.43 0.25  Study year (2016 – 2015) 0.07 ± 0.32 0.05 0.83 |

Each variable was standardised to mean zero and unit variance after log-transformation.

Significant test result (*P* < 0.05) is indicated in bold.

†The residuals of a regression of the log(PTCA) on log(TTCA).

Max VIF = 1.70.

**Appendix 2** General linear model with a binomial distribution explaining survival in relation to wing length, tail length, and pigmentation (i.e. pheomelanin pigmentation, measured as log(TTCA), and eumelanin pigmentation, measured as residual log(PTCA)) in non-breeding Pacific swallows (Sample sizes: males, *n*_survivors_ = 16, *n*_non-survivors_ = 5; females, *n*_survivors_ = 19, *n*_non-survivors_ = 4; *n*_total_ = 44).

| Coefficient ± SE χ^2^ *P* *β* |
| --- |
| log(Wing length) **2.69 ± 0.98 17.76 <0.0001 0.24**  log(Tail length) **−1.71 ± 0.89 5.38 0.02 −0.15**  residual log(PTCA)† **−1.52 ± 0.78 6.52 0.01 −0.13**  log(TTCA) 0.10 ± 0.70 0.02 0.89 0.01 |

Each variable was standardised to mean zero and unit variance after log-transformation.

Selection gradients (*β*) were measured as the averaged gradient vector (Janzen and Stern 1998).

Including sex and its interaction with main terms did not change the results qualitatively (i.e. significant and non-significant terms remain unchanged).

†The residuals of a regression of the log(PTCA) on log(TTCA).

Max VIF = 2.92 (though the variable with highest VIF, tail length, did not change the result qualitatively, in which Max VIF reduced to 1.19).
